# From COP1 Cofactors to Candidate Signaling Hubs: Emerging Roles of SPA Proteins in Plant Growth and Environmental Responses

**DOI:** 10.3390/plants15213326

**Published:** 2026-10-27

**Authors:** Xiaolin Jia, Shaoci Wang, Jie Zhu, Jianping Yang, Shizhan Chen

**Affiliations:** 1 Quality Inspection and Analysis Testing Research Center, Henan Academy of Sciences, Zhengzhou 450046, China; jiaxiaolin@hnas.ac.cn; 2 State Key Laboratory of Wheat and Maize Crop Science, and Center for Crop Genome Engineering, College of Agronomy, Henan Agricultural University, Zhengzhou 450046, China; 3 Henan Plant Natural Products Development Engineering Research Center, Henan Academy of Sciences, Zhengzhou 450003, China

**Keywords:** SPA proteins, E3 ubiquitin ligase, protein kinase, photomorphogenesis, crop improvement

## Abstract

SUPPRESSOR OF PHYA-105 (SPA) proteins bind the E3 ubiquitin ligase CONSTITUTIVELY PHOTOMORPHOGENIC 1 (COP1) and, within the COP1/SPA complex, repress light signaling in plants. For two decades, SPA proteins were regarded mainly as COP1 cofactors. Since 2019, Huq and colleagues have reported that recombinant SPA1 phosphorylates PHYTOCHROME INTERACTING FACTOR 1 (PIF1), PIF4, ELONGATED HYPOCOTYL 5 (HY5) and eukaryotic translation initiation factor 2α (eIF2α) in vitro. Here, we review the SPA family, from its evolution and domain architecture to its regulation by phytochromes, cryptochromes and UVR8, and we grade the evidence for each proposed function as established, reported, inferred or hypothetical. We separate in vitro phosphorylation from in vivo SPA dependence and catalytic validation, and SPA-specific genetic evidence from inferences based on COP1 or HY5. We then relate these mechanisms to de-etiolation, shade avoidance, thermomorphogenesis, photoperiodic flowering, circadian timing and anthocyanin accumulation, and we assess soybean SPA-like alleles that shorten internodes and have been associated with higher yield under dense planting in field trials. We propose, as a working hypothesis, that the SPA subunit is the signal-integrating module of the COP1/SPA complex, and we identify the experiments needed to test this model.

## 1. Introduction

Light is both an energy source and an information signal for plants. Plants sense its quantity, quality, direction and duration with several families of photoreceptors. These include the red/far-red-absorbing phytochromes (phyA–phyE in *Arabidopsis thaliana*), the blue/UV-A-absorbing cryptochromes (CRY1 and CRY2) and the UV-B receptor UV RESISTANCE LOCUS 8 (UVR8). Downstream of these receptors, one of the most influential regulatory modules is the E3 ubiquitin ligase formed by CONSTITUTIVELY PHOTOMORPHOGENIC 1 (COP1) and SUPPRESSOR OF PHYA-105 (SPA) proteins, the COP1/SPA complex [1,2,3]. Seedlings germinating in darkness follow a skotomorphogenic program: the hypocotyl elongates, an apical hook forms and the cotyledons stay closed. Once seedlings reach light, they switch to photomorphogenesis (de-etiolation). COP1 was identified as a genetic locus required to keep dark-grown seedlings in the skotomorphogenic state [4]. Later work showed that COP1 is a RING-type E3 ligase that targets positive regulators of light signaling for degradation by the 26S proteasome [5,6].

The SPA genes were found through a separate genetic route. *spa1* was isolated as a recessive suppressor of the weak *phyA-105* allele [7]. It was soon shown to encode a WD-repeat protein that binds COP1 directly [8,9]. The discovery of three SPA1-like genes, and the constitutive photomorphogenesis of the *spa1 spa2 spa3 spa4* quadruple mutant (*spaQ*) in darkness, placed the SPA family next to COP1 as a core repressor of light signaling [10,11]. The four genes are *SPA1* (AT2G46340), *SPA2* (AT4G11110), *SPA3* (AT3G15354) and *SPA4* (AT1G53090); identifiers for the other genes shown in the tables and figures are listed in Appendix A (Table A1). Much of the literature treats SPA proteins as cofactors, that is, as adaptors that enhance COP1 activity and make it responsive to light [12,13,14].

Three developments now call for a broader view. First, since 2019 one research group (that of E. Huq) and its collaborators have reported, in a series of studies, that SPA1 phosphorylates PIF1, PIF4, HY5 and eIF2α, an activity attributed to its N-terminal domain (NTD) [15,16,17,18]. All of these assays used SPA1, and whether SPA2, SPA3 and SPA4 are active kinases has not been tested. Second, SPA proteins are themselves regulated targets of photoreceptors, and the SPA dependence of phyA and CRY2 turnover [19,20] and differences between the *spaQ* and *cop1* transcriptomes [21] have been taken to suggest partly COP1-independent SPA functions, although this interpretation is not yet secure. Third, allelic variation in soybean SPA-like genes changes internode length, and loss-of-function alleles have been associated with higher grain yield under high-density planting in field trials [22,23].

Earlier reviews covered the COP1/SPA complex and its photoreceptor-mediated inactivation [1,2,3,24], and one review focused on SPA proteins before their kinase activity was reported [25]. This review adds four elements. (i) It appraises the kinase evidence critically and separates in vitro phosphorylation, in vivo SPA dependence and in vivo catalytic validation (Section 4). (ii) It separates functions demonstrated for SPA from functions inferred from COP1 or HY5 data, using explicit evidence criteria. (iii) It integrates bryophyte and crop studies published between 2019 and 2026 (Section 2.4 and Section 6). (iv) It sets out testable criteria for the “hub” model and ranks the experiments that would test it (Section 4.7 and Section 7). Unless stated otherwise, the findings refer to *Arabidopsis thaliana*.

Throughout this review, we use four terms for evidence strength. ‘Established’ denotes findings confirmed by several independent studies; ‘reported’ denotes findings from a single research group or from in vitro assays only, not yet independently replicated; ‘inferred’ denotes SPA roles deduced from COP1 or HY5 data without a direct test of SPA; and ‘hypothesis’ denotes proposed mechanisms that have not been tested directly.

## 2. SPA Protein Family: Evolution, Structure, and Functional Diversification

### 2.1. Discovery of SPA1–SPA4 and Composition of the Family

SPA1 was identified in a screen for extragenic suppressors of *phyA-105*. *spa1 phyA-105* double mutants partially regained responses to low fluence rates of continuous far-red (FR) light, which showed that SPA1 negatively regulates phyA signaling [7]. Cloning of *SPA1* revealed a protein with C-terminal WD-repeats. Its loss increased sensitivity specifically to phyA-perceived light, so it was first described as a phytochrome A-specific signaling component [8]. This view changed once two observations emerged. SPA1 binds COP1 directly through its coiled-coil domain [9], and it also limits light responses under red, blue and white light [26].

*Arabidopsis* encodes three SPA1-like proteins, SPA2, SPA3 and SPA4. SPA3 and SPA4 share about 74% amino acid identity, and their N-terminal domains are about 200 residues shorter than those of SPA1 and SPA2 [10]. SPA3 and SPA4 repress photomorphogenesis in light-grown seedlings under far-red, red and blue light, but single mutants show no phenotype in darkness [10]. Only when all four SPA genes are mutated do dark-grown seedlings undergo strong constitutive photomorphogenesis resembling *cop1* mutants [11]. The four SPA proteins therefore form a partially redundant quartet that, together with COP1, is essential for skotomorphogenesis. They fall into two pairs, SPA1/SPA2 and SPA3/SPA4 [11,27].

### 2.2. N-Terminal Domain, Coiled-Coil Domain and WD40 Repeats

**Figure 1:**
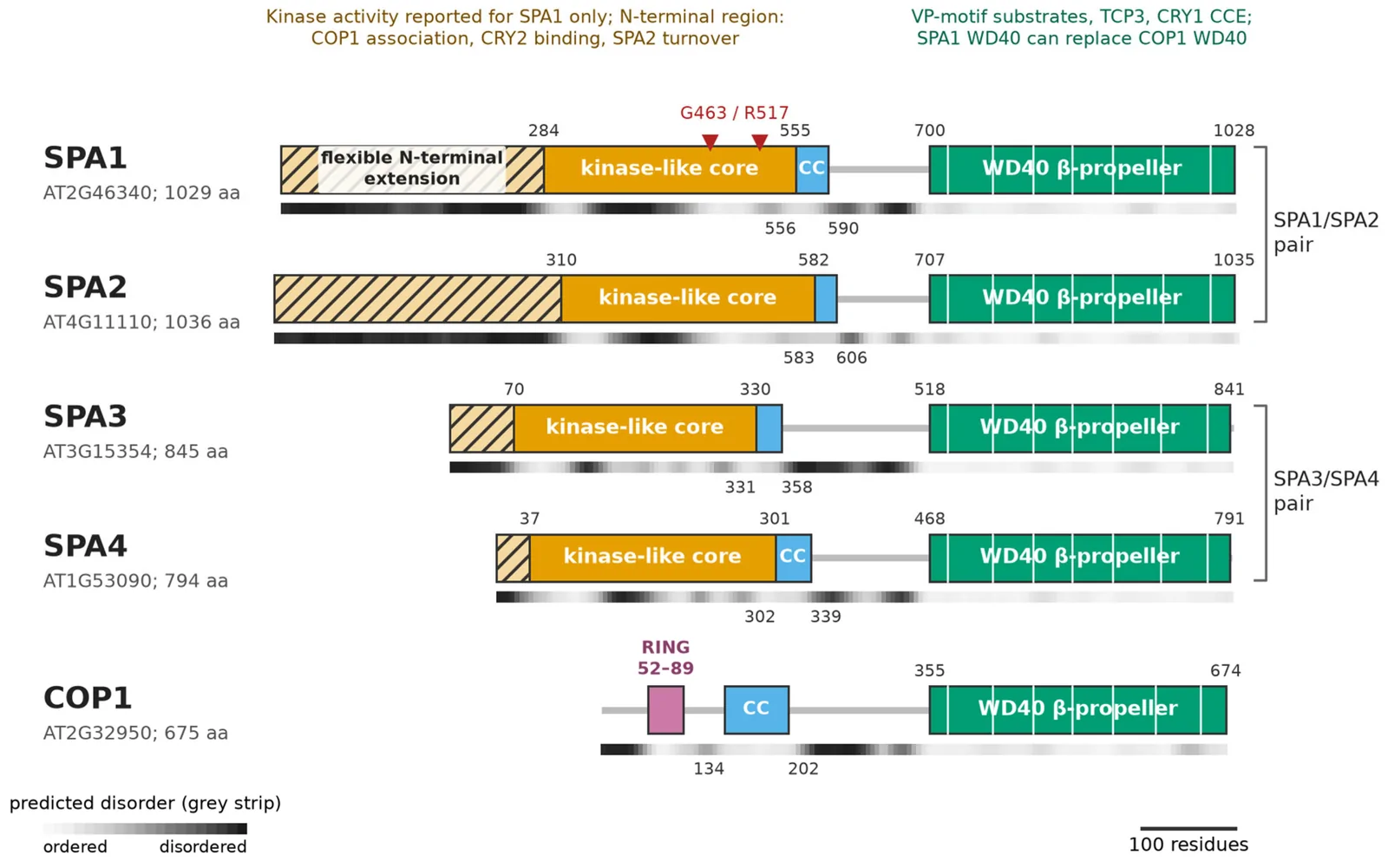
Domain architecture of *Arabidopsis* SPA proteins and COP1. Numbers give the first and last residues of each domain, and AGI codes and protein lengths are shown under the protein names.

All four SPA proteins share a tripartite architecture: an N-terminal domain (NTD), a central coiled-coil (CC) domain [28] and a C-terminal WD40-repeat domain [2,3]. COP1 carries similar CC and WD40 modules but has an N-terminal RING finger in place of the NTD. Figure 1 draws the five proteins to scale and aligns them on the WD40 domain. Two features stand out. The WD40 domains, which form seven-bladed β-propellers, are similar in length in all five proteins (320–329 residues), and so are the kinase-like cores of the four SPA proteins (261–273 residues). The shorter N-terminal regions of SPA3 and SPA4 result mainly from a shorter flexible N-terminal extension, which is predicted to be largely disordered [29] and comprises 283 and 309 residues in SPA1 and SPA2 but only 69 and 36 residues in SPA3 and SPA4. In this review, “NTD” refers to the whole SPA N-terminal region, including the flexible extension and the kinase-like core. We call it a “kinase domain” only where kinase activity has been tested (Section 4).

The three domains connect SPA to different partners. The CC domain links SPA to COP1: the SPA1 CC domain is necessary and sufficient for binding COP1 and contacts the CC domain of COP1 [9]. Through their CC domains, COP1 and SPA proteins form homo- and heterodimers, and endogenous complexes appear to be heterotetramers of two COP1 and two SPA subunits [3,14] that associate further with CULLIN4 (CUL4) and DAMAGED DNA BINDING PROTEIN 1 (DDB1) [30]. We write “COP1/SPA” for the COP1–SPA core and “CUL4–COP1–SPA” for the larger ligase. The SPA1 CC domain does not affect COP1 autoubiquitination, but it promotes COP1-mediated ubiquitination of the MYB factor LAF1 when COP1 concentrations are low [13], so the CC domain both assembles the complex and enhances its activity toward at least one substrate.

The WD40 domain is the main binding platform for substrates and photoreceptors. In COP1, the top face of the β-propeller carries the pocket that binds the valine–proline (VP) motifs of substrates and photoreceptors (Section 3.2) [31]. The SPA1 WD40 domain mediates the blue light-dependent interaction with the CRY1 C-terminal extension (CCE) [32] and binds the transcription factor TCP3 [33]. Both the CC and WD40 domains of SPA1 are required for its repressor function, and overexpressing SPA1 causes hyper-etiolation and lowers the levels of HY5 and LONG HYPOCOTYL IN FAR-RED 1 (HFR1) [34]. In a domain-swap study, the SPA1 WD40 domain largely replaced the WD40 domain of COP1, whereas the COP1 WD40 domain had no activity in the SPA1 context [35]. SPA WD40 domains therefore appear to retain the substrate-recognition capacity of their COP1 counterparts and to have additional SPA-specific properties.

The NTD was long the least understood part of SPA. It has weak similarity to Ser/Thr protein kinases, and for years it was unclear whether it was catalytically active [2]. Genetic analysis nonetheless showed that it is important. Deletions and missense mutations in the SPA1 NTD strongly impaired SPA1 activity in darkness, but light responsiveness remained intact. The larger N-terminal region contributes to formation of the COP1/SPA complex, although deleting only the kinase-like core did not abolish binding to COP1 or cryptochromes [36]. Blue light-dependent binding of CRY2 requires the SPA1 N-terminus [37], so the N-terminal half, not only the WD40 domain, provides a photoreceptor interface. Domain swaps between SPA1 and SPA2 showed that the NTD largely determines the paralog-specific light responsiveness of these two proteins [38], and the NTD of SPA2 is needed for its light-induced degradation [39]. The NTD is therefore a multifunctional module involved in SPA activity, complex formation and protein turnover. Consequently, the phenotype of an NTD variant designed to impair kinase activity may also reflect effects on these other functions (Section 4.2). In summary, COP1 binds SPA through the CC domain, substrates and CRY1 bind through the WD40 domain, and CRY2 binding, paralog-specific light responsiveness and turnover depend on the NTD. This modular arrangement is the structural basis for asking whether signals enter the COP1/SPA complex through its SPA subunits (Section 4.7).

### 2.3. Functional Redundancy and Specificity of SPA1–SPA4

Redundancy within the SPA family depends on developmental stage, light condition and tissue (Table 1). Three mechanisms account for most of the specificity. The first is differential expression. All four endogenous SPA proteins form stable complexes with COP1 in light and in darkness, but they differ in expression across tissues and light conditions [14]. The second is paralog-specific protein stability. SPA2 is a strong repressor in darkness but loses most of its function once seedlings are exposed to light, when it is rapidly degraded [40], although it retains some activity under several light conditions [41]. SPA1 is a potent repressor in both light- and dark-grown seedlings [40], and SPA3 and SPA4 are stable in light [39]. These differences explain why SPA1 and SPA2 dominate in dark-grown seedlings, whereas SPA3 and SPA4 contribute little in darkness, repress photomorphogenesis in light-grown seedlings and dominate the control of elongation growth in adult plants [10,27]. The third mechanism is tissue context. Expressing SPA1 only in phloem companion cells is sufficient to rescue the *spa1* phenotype of dark-grown seedlings and to restore normal flowering time, whereas full rescue of leaf expansion requires SPA1 in both phloem and mesophyll. SPA1 in these tissues affects cell division and expansion in the epidermis, so its effects are partly non-cell-autonomous [42].

Paralog specificity concerns differences among SPA proteins. A separate question is what the SPA family as a whole contributes beyond COP1, and the genetic answer is clear but limited. *cop1* null mutants arrest at the seedling stage, whereas *spaQ* mutants complete their life cycle, which indicates that COP1 retains some activity without SPA proteins [47]. Conversely, transcriptome profiling of dark-grown *spaQ* seedlings found more than 7200 differentially expressed genes, which overlap substantially with those deregulated in *cop1* [21]. Most SPA functions therefore appear to be exerted together with COP1. Whether some are independent of COP1 is discussed in Section 4.7.

### 2.4. Conservation and Divergence of SPA in Bryophytes, Monocots and Dicots

COP1 exists in both plants and animals. In humans it is a key regulator of tumorigenesis [3], and structural work on the human COP1 WD40 domain has shown how it recognizes substrates [48]. SPA proteins, by contrast, have been found only in plants. A comparative study expressed COP1 and SPA orthologs from the moss *Physcomitrella* (now *Physcomitrium*) *patens* and from rice in *Arabidopsis* mutants. COP1 function was well conserved across these species, whereas SPA function had diverged considerably [49]. Rice OsSPA1, for example, rescued the *spa* mutant phenotype of dark-grown seedlings but not that of light-grown seedlings or adult plants [49]. COP1 is a single-copy gene in *Arabidopsis* and rice, although duplicates exist in paleopolyploid crops such as soybean [22]. SPA proteins, in contrast, form small families of two to four members [49]. This pattern is consistent with sub- or neofunctionalization of SPA paralogs after gene duplication.

Studies in bryophytes support this interpretation. In *P. patens*, double mutants of the two SPA genes (*PpSPAa* and *PpSPAb*) have smaller gametophores in light and continue to grow in darkness, yet they etiolate normally [50]. The moss COP1/SPA module therefore controls growth, but it does not control the dark-to-light developmental switch in the way it does in *Arabidopsis*. Later work showed that COP1, SPA and cryptochrome cooperate in light signaling and photomorphogenesis in *P. patens* [51]. In the liverwort *Marchantia polymorpha*, MpSPA and MpCOP1 both interact with MpHY5, and MpSPA promotes MpCOP1-mediated ubiquitination of MpHY5. Unlike *Arabidopsis* CRY, however, MpCRY does not inhibit this ubiquitination [52]. The core COP1/SPA–HY5 module therefore seems to predate seed plants. Its tight coupling to cryptochrome-mediated inactivation, and its control of skotomorphogenesis, may have evolved later.

In angiosperms, SPA genes have been characterized in rice, maize and soybean (Table 2). Maize ZmSPA genes modulate photomorphogenesis and increase plant height when expressed in *Arabidopsis* [53]. In soybean, the WD40 protein PH13 and its paralog Glyma.12G224600, named PHP in one study and RIN1/GmSPA3a in another, control internode elongation (Section 6) [22,23]. None of these studies has tested kinase activity of a non-Arabidopsis SPA protein.

## 3. Canonical COP1/SPA Signaling and Its Regulation by Photoreceptors

### 3.1. Formation and Activity of the COP1/SPA Complex

In darkness, COP1 accumulates in the nucleus. There, it ubiquitinates photomorphogenesis-promoting transcription factors and targets them for proteasomal degradation [5,6]. SPA proteins are needed for full COP1 activity, and *spaQ* largely phenocopies *cop1* in darkness [11]. Biochemically, SPA1 alters the E3 activity of COP1 toward HY5 in vitro [12] and stimulates LAF1 ubiquitination when COP1 is limiting [13]. The CUL4–COP1–SPA ligase regulates photomorphogenesis and flowering time [30] and is required for the light-induced degradation of PIF1 [54], and PIF1 in turn enhances the E3 ligase activity of COP1 [55]. SPA proteins also help control where COP1 is located. Light excludes COP1 from the nucleus, and this exclusion requires SPA proteins; SPA proteins do not, however, affect COP1 nuclear accumulation in darkness [56].

### 3.2. Substrate Recognition and Ubiquitin-Mediated Proteolysis

Most COP1 substrates carry short valine–proline (VP) motifs that bind a conserved pocket on the top face of the COP1 WD40 β-propeller, the ring-shaped fold formed by the WD40 repeats. Crystal structures of human COP1 with a Trib1 peptide [48], and of Arabidopsis COP1 with VP peptides from HY5 and UVR8 [31], define this binding mode. Because photoreceptors also carry VP motifs, they compete with substrates for the same site (Section 3.3) [31]. A cryo-electron microscopy (cryo-EM) structure of UV-B-activated UVR8 bound to COP1 was obtained from a reconstituted COP1–SPA4–UVR8 complex. It resolved the UVR8 core, the UVR8 VP motif and the COP1 WD40 domain, but not SPA4 [57].

Table 3 lists representative COP1/SPA targets and scores three types of SPA-specific evidence separately. Physical interaction with an SPA protein shows that a target can engage the complex through SPA. Altered protein level or turnover in *spa* mutants shows a genetic requirement for SPA, which may be indirect. Ubiquitination assays that include an SPA protein show that SPA takes part in the modification biochemically. Only PIF1 and HY5 are supported by all three types of evidence; CO, PIF5, phyA and CRY2 are supported by two, and HFR1, LAF1, COL12, TCP3, PAP1/PAP2, PIF4 and phyB by one. For HYH, BBX21, BBX22, BBX28 and several further targets, only COP1 involvement has been shown.

TCP3 is notable because it lacks a VP motif. Its TCP domain binds the WD40 domains of both COP1 and SPA1, although binding still requires the VP-binding cleft of COP1 [33]. Several other COP1 targets have been identified through COP1 alone. They include DELLA proteins, the stomatal regulators ICE1/SCRM, GIGANTEA (GI; together with ELF3) and the chromatin regulator VERNALIZATION INSENSITIVE 3-LIKE 1 (VIL1) [58,59,60,61]. Whether SPA proteins are needed for their turnover has not been tested.

The context dependence of PIF regulation deserves emphasis. CUL4–COP1–SPA degrades PIF5 in light but stabilizes it at night [62]. COP1 also stabilizes PIF5 in light-grown plants, and UVR8 disrupts this effect [63]. In darkness, COP1/SPA stabilizes PIF3 indirectly by restraining the kinase BIN2 [64]. The same complex can therefore destabilize or stabilize a given PIF depending on time of day, light quality and cofactors.

**Table 3:** Representative COP1/SPA targets in *Arabidopsis* and the SPA-specific evidence for each. Mechanism: E3, ubiquitination substrate; K, reported SPA1 kinase substrate; NC, noncanonical (nonproteolytic) regulation. SPA-specific evidence is scored separately for physical interaction with an SPA protein (binding), altered protein level or turnover in *spa* mutants (genetic) and ubiquitination assays that included an SPA protein (Ub assay). +, shown; –, not shown in the cited studies; NA, not applicable. Targets with “–” in all three columns are COP1 targets for which an SPA requirement has not been tested. AGI codes are given in parentheses.

Target	Class	Mechanism	Binding	Genetic	Ub Assay	Physiological Output	Refs.
HY5 (AT5G11260)	bZIP TF	E3, K	+	+	+ (in vitro)	De-etiolation; pigment genes; root growth	[5,12,17,65]
HYH (AT3G17609)	bZIP TF	E3	–	–	–	De-etiolation	[66]
HFR1 (AT1G02340)	bHLH TF	E3	–	+	–	FR/blue de-etiolation	[26]
LAF1 (AT4G25560)	MYB TF	E3	–	–	+ (in vitro; SPA1 CC domain)	FR responses	[13]
BBX21 (AT1G75540), BBX22 (AT1G78600), BBX28 (AT4G27310)	BBX TFs	E3	–	–	–	De-etiolation	[67,68,69,70]
CO (AT5G15840)	BBX TF	E3	+	+	–	Photoperiodic flowering	[44,71,72]
COL12 (AT3G21880)	BBX TF	E3	+	–	–	Flowering time; architecture	[73]
TCP3 (AT1G53230)	TCP TF (no VP motif)	E3	+	–	–	Anthocyanin; flowering time	[33]
PAP1 (AT1G56650), PAP2 (AT1G66390)	MYB TFs	E3	+	–	–	Anthocyanin	[74]
PIF1 (AT2G20180)	bHLH TF	E3, K	+	+	+ (in vivo)	De-etiolation	[15,54,75]
PIF4 (AT2G43010)	bHLH TF	K	–	+	–	Thermomorphogenesis	[16]
PIF5 (AT3G59060)	bHLH TF	E3	–	+	+ (in vivo)	Diurnal growth; UV-B inhibition of elongation	[62,63]
PIF3 (AT1G09530)	bHLH TF	NC: COP1/SPA restrains BIN2	NA	NA	NA	Skotomorphogenesis	[64]
eIF2α (AT5G05470)	Translation factor	K	–	NA	NA	Translation efficiency	[18]
phyA (AT1G09570)	Photoreceptor	E3	+	+	–	phyA desensitization	[19,76,77]
phyB (AT2G18790)	Photoreceptor	E3	+	–	–	phyB turnover	[78,79]
CRY2 (AT1G04400)	Photoreceptor	E3	+	+	–	CRY2 turnover	[20,37]
DELLA proteins	GRAS regulators	E3	–	–	–	Growth	[58]
ICE1/SCRM (AT3G26744)	bHLH TF	E3	–	–	–	Stomatal development	[59]
GI (AT1G22770)	Clock protein	E3	–	–	–	Circadian clock; flowering	[60]
VIL1 (AT3G24440)	PHD-finger chromatin regulator	E3	–	–	–	Light-dependent chromatin remodeling	[61]

### 3.3. Regulation by Phytochromes, Cryptochromes and UVR8

**Figure 2:**
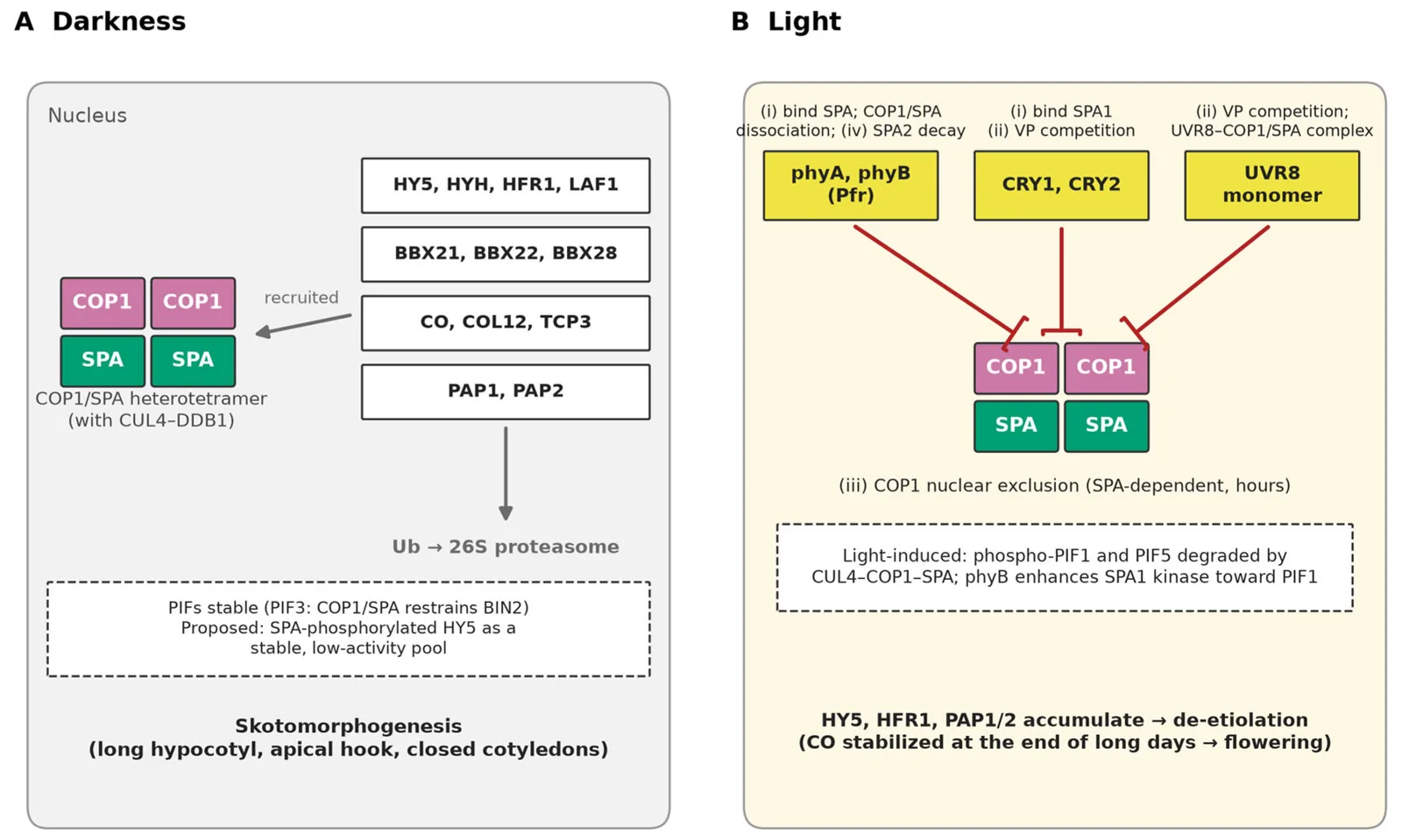
Canonical regulation of COP1/SPA by light. (**A**) In darkness, nuclear COP1/SPA heterotetramers, which can associate with CUL4–DDB1, recruit photomorphogenesis-promoting transcription factors (gray arrow) and ubiquitinate them (Ub) for degradation by the 26S proteasome. The substrates shown are HY5, HYH, HFR1, LAF1, BBX21, BBX22, BBX28, CO, COL12, TCP3, PAP1 and PAP2 [5,13,26,33,44,66,67,68,69,73,74]. PIFs are stable, and PIF3 is protected because COP1/SPA restrains BIN2 [64]. SPA-dependent phosphorylation has been proposed to maintain a stable, low-activity HY5 pool [17]. Seedlings follow skotomorphogenesis. (**B**) In light, photoreceptors inactivate COP1/SPA (red flat-headed lines) by the four mechanisms described in Section 3.3. (i) Active (Pfr) phytochromes and cryptochromes bind SPA proteins and weaken the COP1/SPA interaction [32,77,79,80]. (ii) Cryptochromes and UVR8 compete with substrates for COP1 through VP motifs, and UVR8 forms UVR8–COP1/SPA complexes [31,81,82,83]. (iii) Over hours, COP1 is excluded from the nucleus in an SPA-dependent manner [56]. (iv) Phytochromes trigger SPA2 degradation [39,45]. In light, phosphorylated PIF1 and PIF5 are degraded by CUL4–COP1–SPA, and phyB enhances SPA1-mediated PIF1 phosphorylation [15,54,62]. HY5, HFR1 and PAP1/2 accumulate and drive de-etiolation. CO is stabilized at the end of long days and promotes flowering [71]. Pfr, far-red-absorbing active phytochrome; VP, valine–proline motif.

Light inactivates COP1/SPA mainly by acting on the complex itself rather than on its substrates. Four mechanisms have been described (Figure 2) [1,3]. They differ in which subunit the photoreceptor engages and in how quickly they act, and two of them work through SPA proteins.

The first mechanism disrupts the COP1–SPA interaction (Figure 2B(i)), and here the photoreceptors dock on the SPA subunit. Blue light-activated CRY1 binds SPA1, through the CRY1 CCE and the SPA1 WD40 domain, and weakens the COP1–SPA1 association [32,80]. Paradoxically, the blue light-induced interaction of CRY1 with COP1 itself requires SPA proteins [84]. This suggests that CRY1 engages and remodels the whole complex, rather than simply pulling SPA1 away from COP1. CRY2 also interacts with SPA1 in a blue light-dependent manner, through the SPA1 N-terminus, and SPA1 acts genetically downstream of CRY2 to suppress COP1-dependent CO degradation [37]. Phytochromes use a comparable strategy. Light-activated phyA and phyB bind SPA proteins, and SPA1 binds phyA only in its active Pfr form, thereby reorganizing the COP1/SPA complex [77]. Red light-activated phyB binds SPA1 and represses its association with COP1 [79].

The second mechanism acts on COP1 rather than on SPA. Photoreceptors carry VP motifs that compete with substrates for the WD40 pocket of COP1 (Figure 2B(ii)). CRY2 uses a VP motif in its C-terminal domain to compete with substrates for COP1 binding [81]. CRY1 uses a similar VP motif to displace substrates from COP1, and this motif is required for de-etiolation and anthocyanin accumulation in blue light [82]. UV-B-activated UVR8 monomers bind COP1 cooperatively through their VP motif and their photosensing core, which prevents COP1 from binding HY5 [31,85,86]. UV-B also separates the COP1/SPA core from CUL4–DDB1 and promotes the formation of UVR8–COP1–SPA complexes. This switch turns COP1 from a repressor into a promoter of UV-B photomorphogenesis [83]. Whether SPA is required for UVR8 action has not been established.

The two remaining mechanisms are slower and change the location or the amount of the complex. Prolonged light gradually moves COP1 out of the nucleus, and this exclusion depends on SPA proteins (Figure 2B(iii)) [56]. Light also reduces SPA protein levels, most strongly for SPA2 (Figure 2B(iv); Section 3.4) [40,45]. The four mechanisms therefore probably act on different time scales. Photoreceptor binding to SPA and VP-motif competition require only photoreceptor activation, SPA2 degradation occurs within minutes [45], and COP1 nuclear exclusion takes hours [56]. Direct in vivo kinetics of VP-motif displacement are not yet available, so this time-scale hierarchy remains a hypothesis.

Phytochrome effects on SPA1 are not uniform. Under FR light, phyB interacts with SPA1, increases its nuclear accumulation and thereby represses photomorphogenesis [87]. Under blue light, phyA and phyB act synergistically to repress SPA1 [88]. These apparently opposite effects may be reconciled if the outcome depends on which SPA-containing subcomplex a photoreceptor engages, and on whether the photoreceptor itself is active; under FR, phyB is largely in the inactive Pr form. Testing this idea will require the stoichiometry of photoreceptor–SPA–COP1 complexes to be quantified under defined light conditions.

### 3.4. Light-Dependent Reorganization and Turnover of SPA Proteins

SPA proteins are regulated targets as well as regulators. When dark-grown seedlings are exposed to light, SPA2 is degraded within 5–15 min, SPA1 more weakly, and COP1 not at all [40,45]. Rapid SPA2 degradation requires COP1 and phytochromes; cryptochromes are not needed for the rapid decrease in blue light [45]. A later study showed that SPA2 degradation requires both its NTD and its DDB1-interacting WD40 domain. In far-red and blue light, SPA2 is destabilized exclusively by phyA, whereas SPA3 and SPA4 are stable in light [39]. Domain-swap experiments trace the different light responsiveness of SPA1 and SPA2 to the NTD [38]. The NTD thus couples at least SPA1 and SPA2 to paralog-specific modes of light-triggered inactivation.

SPA abundance is also shaped by gene expression and the circadian clock. The four SPA genes differ in their expression across tissues and light conditions [14]. The bHLH factor MYC2 binds a G-box in the *SPA1* promoter and regulates *SPA1* expression in a COP1-dependent manner [89]. Under constant light, SPA1 protein levels oscillate with a circadian rhythm [90], a point discussed further in Section 5.4. SPA levels therefore reflect light history, time of day and hormone signaling, not only the current light environment.

## 4. Beyond Ubiquitination: Reported Kinase Activity of SPA1

### 4.1. Discovery of SPA1 Kinase Activity

As noted in Section 2.2, whether the NTD is catalytically active or a pseudokinase (a kinase-like fold without catalytic activity) was long uncertain [2]. In 2019, Paik and colleagues reported that recombinant SPA1 phosphorylates PIF1 in vitro, that SPA proteins are required for light-induced PIF1 phosphorylation in vivo, and that plants expressing SPA1^R517E^ are defective in light-induced PIF1 degradation [15]. This substitution targets a conserved Glu–Arg salt bridge of eukaryotic protein kinases and reduces, but does not abolish, SPA1 kinase activity in vitro [15]. The same variant, like SPA1^G463V^, had earlier failed to rescue the dark phenotype of *spaQ*, although its protein accumulated to wild-type levels [36]. The same group later reported SPA-dependent phosphorylation of PIF4, HY5 and eIF2α [16,17,18]. On this basis, the authors proposed that COP1/SPA complexes are “a cognate kinase–E3 ligase complex” that phosphorylates its substrates and then targets them for removal [15].

**Figure 3:**
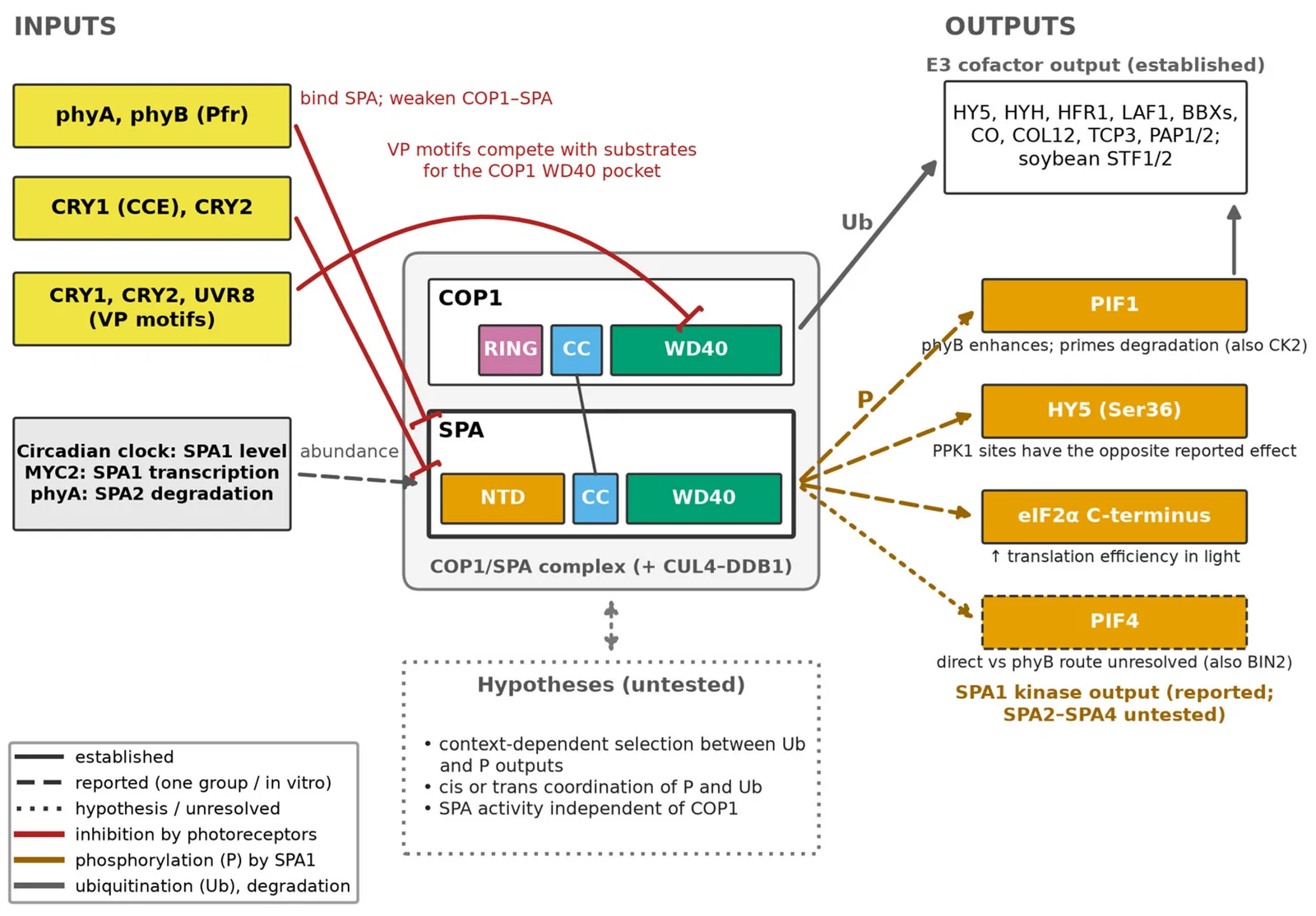
A working model of the SPA subunit as the signal-integrating module of the COP1/SPA complex. Line style indicates the level of evidence: solid, established; dashed, reported (a single research group and/or in vitro only); dotted, hypothesis.

The COP1/SPA complex is treated as the signaling unit, and the model asks which inputs and outputs act through its SPA subunit (Figure 3). Active phytochromes and cryptochromes bind SPA proteins and weaken the COP1–SPA interaction [32,37,77,79,80], whereas the VP motifs of CRY1, CRY2 and UVR8 compete with substrates for the WD40 pocket of COP1 [31,81,82]. SPA abundance is regulated by the circadian clock (SPA1 protein), by MYC2 (SPA1 transcription) and by phyA-triggered, NTD-dependent degradation of SPA2 [39,89,90]. The established output is COP1 cofactor activity, through which the complex ubiquitinates (Ub) and degrades HY5, HYH, HFR1, LAF1, BBX proteins, CO, COL12, TCP3, PAP1/2 and, in soybean, STF1/2 (Table 3) [22,23]. The second reported output is phosphorylation (P) by SPA1, which has been attributed to the NTD; whether SPA2–SPA4 have kinase activity has not been tested. phyB enhances SPA1-mediated phosphorylation of PIF1, which is then degraded by CUL4–COP1–SPA [15,54]; CK2 also phosphorylates PIF1 [75]. SPA-dependent phosphorylation of HY5 at Ser36 has been proposed to produce a stable, less active form [17,91], whereas PPK1 phosphorylation at Thr55, Ser56 and Ser60 increases COP1 binding [65]. SPA1 phosphorylates the eIF2α C-terminus and increases translation efficiency in light [18]. SPA proteins are required for PIF4 accumulation at warm temperature, but a direct kinase effect has not been separated from a phyB-mediated route, and BIN2 also phosphorylates PIF4 [16,92].

### 4.2. How Strong Is the Evidence for SPA1 Kinase Activity?

Because the dual-activity model depends on SPA1 kinase activity, we evaluated the evidence for this activity at three levels (Table 4). Level 1 is phosphorylation of a substrate by recombinant SPA1 in vitro. Level 2 is in vivo SPA dependence, meaning that phosphorylation of the substrate is reduced in *spaQ*; it shows that SPA proteins are required, directly or indirectly. Level 3 is in vivo catalytic validation, in which an SPA1 variant that is catalytically impaired but otherwise intact fails to restore phosphorylation or function in *spaQ*.

The three levels describe the type of evidence available for each substrate (Table 4). Three limitations qualify this evidence. We discuss them in the order of the levels to which they apply and end with one that applies to all levels.

First, Level 1 assays require controls against co-purifying kinases, such as catalytically inactive variants purified in parallel, because the NTD is only weakly similar to canonical Ser/Thr kinases [2]. Structural data would settle whether the NTD adopts an active kinase conformation.

Second, Level 3 depends on the kinase-impaired variant. The best-characterized of these is SPA1^R517E^, the kinase-impaired variant described in Section 4.1, which reduces but does not abolish SPA1 kinase activity in vitro [15]. This and other mutations in the NTD impair SPA1 activity, and the N-terminal region contributes to COP1 association and controls SPA turnover (Section 2.2) [36,38,39]. A variant that fails to complement *spaQ* therefore does not by itself show that catalysis, rather than a structural role, is responsible. The decisive controls are the following. The variant should fold correctly and bind COP1 and photoreceptors normally. Its protein stability in light should match that of wild-type SPA1. Ideally, several independent catalytic-site substitutions should give concordant results. These controls have been reported only in part. SPA1^R517E^ accumulates to wild-type levels [36], but we found no report of its COP1-binding capacity.

Third, and independently of the level reached, no independent laboratory has yet reproduced SPA1 kinase activity with purified protein. Independent replication, ideally with SPA1 expressed in a heterologous system and purified to homogeneity, would considerably strengthen the model.

Taken together, SPA proteins are required for phosphorylation of PIF1, HY5 and eIF2α in vivo (Level 2), and recombinant full-length SPA1 phosphorylates all four substrates in vitro (Level 1). For PIF4, phosphorylation by SPA1 is supported only at Level 1. The in vivo data show SPA-dependent regulation of PIF4 accumulation, which may be direct or mediated by phyB (Section 4.3), rather than SPA-dependent PIF4 phosphorylation. The assignment of catalysis to the NTD rests on sequence similarity and on the R517E substitution, not on assays with the isolated domain. Neither a clean separation of catalytic from structural NTD functions (Level 3) nor independent replication has yet been achieved. We therefore classify SPA1 kinase activity as reported: it is supported at Levels 1 and 2 for SPA1 but has not been independently replicated or catalytically validated, and it is untested for SPA2–SPA4.

### 4.3. SPA1-Mediated Phosphorylation of PIF1 and PIF4

PIFs are bHLH transcription factors that together repress photomorphogenesis in darkness [95]. On light exposure, PIF1 is rapidly phosphorylated, ubiquitinated and degraded, and SPA proteins are required for all three steps [15]. The CUL4–COP1–SPA complex preferentially binds phosphorylated forms of PIF1 [54]. Photoactivated phyB binds SPA1 through the phyB C-terminus and enhances the recruitment of PIF1 for phosphorylation [15]. SPA1 is not the only PIF1 kinase, however. CK2 phosphorylates PIF1 at seven Ser/Thr residues, and a phospho-null PIF1 variant is degraded more slowly in light [75]. The PHOTOREGULATORY PROTEIN KINASES (PPKs) mediate phytochrome-induced phosphorylation of PIF3 [93], and phytochrome itself has been proposed to phosphorylate PIFs [94]. Whether SPA1, CK2 and PPK sites on PIF1 overlap, act in sequence, or are redundant has not been examined. A plausible model is that SPA1 contributes light-specific priming within the ligase complex, on top of constitutive CK2 phosphorylation. Testing this model would require site-resolved phosphoproteomics in *spaQ*, *ck2* and *ppk* backgrounds.

At warm ambient temperature, PIF4 is the central growth regulator (Section 5.3) [96]. *spaQ* seedlings are thermo-insensitive, and SPA1 phosphorylates PIF4 in vitro, but SPA-dependent phosphorylation of PIF4 has not been shown in vivo. A kinase-impaired SPA1 variant fails to restore PIF4 levels or thermoresponsive growth in *spaQ* [16]. The same study reported that phyB, a negative regulator of PIF4, is stabilized in *spaQ* at warm temperature, so the authors proposed that SPA proteins regulate the phyB–PIF4 module at more than one level [16]. The current data thus cannot distinguish direct stabilization of PIF4 by phosphorylation from an indirect effect through phyB. BIN2 also phosphorylates PIF4, which lowers its stability; brassinosteroids relieve this by inhibiting BIN2 [92]. The decisive experiments would be to express the kinase-impaired SPA1 in a *spaQ phyB* background and to map SPA-dependent PIF4 phosphosites in vivo.

### 4.4. Phosphorylation and Regulation of HY5

HY5 exists in phosphorylated and unphosphorylated isoforms produced by phosphorylation within its COP1-binding domain. The phosphorylated form interacts less strongly with COP1 and is more stable, but it binds target promoters less well and is physiologically less active [91]. Hardtke et al. mapped this modification to Ser36, analyzed a non-phosphorylatable HY5^S36A^ variant, and attributed the phosphorylation to a light-regulated kinase activity [91], which later literature often described as CK2-like. Wang et al. later reported that SPA1 phosphorylates HY5 at Ser36 in vitro and that phosphorylated HY5 is absent from *spaQ* in vivo. Unphosphorylated HY5 interacted more strongly with COP1 and SPA1 and was preferentially degraded, whereas phosphorylated HY5 was more stable in darkness [17].

A 2024 study by Zhang et al. [65] complicates this picture. It showed that PPK1 phosphorylates HY5 at Thr55, Ser56 and Ser60. This phosphorylation increases HY5 binding to BBX24, which represses HY5 activity, and to COP1, which promotes HY5 degradation. PPK1 phosphorylation therefore makes HY5 both less active and less stable [65]. SPA-dependent phosphorylation (reported at Ser36 [17]) and PPK1-dependent phosphorylation thus have opposite reported effects on HY5–COP1 binding. They target different residues, which is consistent with a site-specific phospho-code in which each site is read differently by COP1. How the two kinases are coordinated in time, and whether they act on the same HY5 pool, is the key open question. Until this question is resolved, the attractive idea that SPA-dependent phosphorylation maintains a stable, low-activity HY5 reserve in darkness should be regarded as a hypothesis.

### 4.5. SPA1–eIF2α Signaling and Translational Control

The most unexpected reported SPA substrate is not a transcription factor but a core component of the translation machinery. *spaQ* seedlings show lower translation efficiency after light exposure, and SPA1 phosphorylates several Ser/Thr residues in the C-terminal region of eIF2α under light conditions [18]. These sites differ from Ser52, the well-studied eIF2α site whose phosphorylation inhibits translation in other eukaryotes. Phosphorylation of the eIF2α C-terminus increases translation efficiency and promotes photomorphogenesis, and a non-phosphorylatable C-terminal variant reduces translation efficiency [18].

This finding should be placed next to an established light-to-translation route. During de-etiolation, phyA and cryptochromes inactivate COP1, which activates an auxin–TARGET OF RAPAMYCIN (TOR) pathway that phosphorylates RIBOSOMAL PROTEIN S6 (RPS6) and enhances translation [97]. SPA1–eIF2α and COP1–TOR–RPS6 may therefore represent parallel or interacting branches through which the COP1/SPA module controls translation. The finding has two further implications. First, SPA proteins have at least one reported pro-photomorphogenic function in the light, which does not fit a model in which SPA proteins act only as light-inactivated repressors. Second, we found no report testing whether eIF2α phosphorylation requires COP1.

### 4.6. Coordination Between Phosphorylation and Ubiquitination

The reported substrates suggest that SPA-dependent phosphorylation can have at least three outcomes. For PIF1, phosphorylation acts as a degradation signal. It increases recognition by CUL4–COP1–SPA and speeds degradation [15,54], together with phosphorylation by other kinases such as CK2 [75]. For HY5, SPA-dependent phosphorylation has been reported to have the opposite effect, lowering COP1 binding and stabilizing HY5 at the cost of lower activity [17,91]. This category is provisional, because PPK1-dependent phosphorylation at other residues increases COP1 binding [65]. PIF4 is not assigned to either category, because a direct effect of phosphorylation on its stability has not been separated from the phyB route (Section 4.3). For eIF2α, phosphorylation changes the activity of a target that COP1 is not known to degrade [18]. Which outcome occurs probably depends on the position of the phosphorylated residues relative to VP motifs or other degradation signals (degrons), on the presence of other kinases and E3 ligases, and on photoreceptor state. CO illustrates the open questions. CO is phosphorylated, the phosphorylated form is relatively more abundant in light, and this ratio depends on COP1 [98]. SPA1 binds CO directly [44], but whether SPA1 phosphorylates CO has not been tested.

A second question is where the two activities meet. Kinase and E3 activities could be coordinated in cis, when an SPA protomer phosphorylates a substrate that the neighboring COP1 protomer then ubiquitinates. They could also act in trans, if SPA proteins act as kinases outside COP1/SPA complexes. These possibilities could be distinguished with SPA variants that retain kinase activity but cannot bind COP1 (for example CC-domain mutants), and with kinase-impaired variants that retain COP1 binding, each expressed in *spaQ*.

### 4.7. Is SPA a Signaling Hub? Separating the SPA Subunit from the COP1/SPA Complex

Because SPA proteins act within COP1/SPA, we apply each hub criterion from Section 1 to the SPA subunit and ask whether the relevant mechanism requires COP1.

The first criterion, multiple independent inputs, is met at the level of the SPA subunit. phyA and phyB bind SPA proteins [77,79], CRY1 binds the SPA1 WD40 domain through its CCE [32,80], CRY2 binds the SPA1 N-terminus [37], and the CRY1–COP1 interaction itself requires SPA [84]. SPA proteins are also the regulated component of the complex. Phytochromes trigger NTD-dependent degradation of SPA2, whereas COP1 is not degraded in light [39,40,45]; SPA1 protein levels are under circadian control [90]; and MYC2 regulates *SPA1* transcription [89]. Not all inputs act through SPA, however, because VP-motif competition by CRY1, CRY2 and UVR8 targets the WD40 domain of COP1 [31,81,82]. The complex therefore receives light signals through both subunits, but phytochrome and cryptochrome binding, paralog-specific turnover and circadian control of abundance act specifically on SPA.

The second criterion, the presence of more than one biochemical output, is met only provisionally and only for SPA1. The first output, COP1 cofactor activity, is established and is exerted within the complex (Section 3). The second, kinase activity, is reported for SPA1 alone and is subject to the caveats in Section 4.2. SPA-dependent turnover of phyA and CRY2 [19,20] is best counted as a target of the cofactor output. It would count as a separate output only if a COP1-independent mechanism were shown.

The third criterion, context-dependent output selection, is not demonstrated and remains a hypothesis. The best-documented case is coupling rather than switching. In light, phyB enhances SPA1-mediated PIF1 phosphorylation, and phosphorylated PIF1 is then degraded by CUL4–COP1–SPA [15,54]. Light therefore appears to change substrate choice within a coupled kinase–E3 unit rather than to switch between outputs: most transcription-factor substrates are stabilized in light, whereas phosphorylated PIF1 and PIF5 are degraded. Three further observations are consistent with output selection but do not demonstrate it. eIF2α phosphorylation is a light-phase output with no known link to degradation [18]. HY5 carries SPA-dependent and PPK1-dependent phosphosites with opposite reported effects [17,65]. At warm temperature, SPA1 kinase activity appears to act through PIF4 in shoots and through HY5 in roots [46]. Demonstrating selection would require measuring the kinase and E3 outputs of the same SPA pool under the same conditions.

Evidence that SPA acts without COP1 is limited. The strongest case is phyA degradation. In the viable *cop1-4* and *cop1-6* mutants, light-induced phyA degradation was affected only when metabolizable sugar was present, whereas it was strongly attenuated in *spa1 spa2 spa3* regardless of sucrose [19]. CRY2 degradation in blue light is reduced in all *spa* mutants tested, whereas several viable *cop1* mutants reduce but do not abolish it [20]. Both comparisons have an important caveat. Because *cop1* null alleles are seedling-lethal, turnover studies rely on hypomorphic *cop1* alleles, whereas *spaQ* lacks all SPA function. A weaker or conditional effect in *cop1* is therefore also expected from residual COP1 activity, and neither result demonstrates a COP1-independent SPA activity. Other observations are often cited in this context but do not bear on the question. The viability of *spaQ*, compared with the lethality of *cop1*, shows that COP1 can act without SPA, not the reverse [47]. Divergence of SPA function in moss reflects evolutionary lability rather than COP1 independence [49,50], and differences between the *spaQ* and *cop1* transcriptomes [21] may reflect differences in mutant severity or residual COP1 activity. The COP1 requirement for SPA-dependent phosphorylation of eIF2α and HY5 has not been tested. COP1-independent SPA functions are therefore possible but not yet demonstrated. Formal proof will require SPA variants that cannot bind COP1 but retain kinase activity, expressed in *spaQ*.

What, then, qualifies SPA, rather than the complex, as a candidate hub? On current evidence, the COP1/SPA complex is the unit that integrates light signals and executes most outputs. Three features nevertheless place the integrating steps in its SPA subunit. Phytochrome and cryptochrome inputs dock on SPA domains; SPA abundance, unlike that of COP1, is regulated by light, the clock and paralog-specific turnover; and the second reported biochemical output, kinase activity, resides in SPA1.

## 5. SPA Proteins in Plant Growth and Environmental Responses

**Figure 4:**
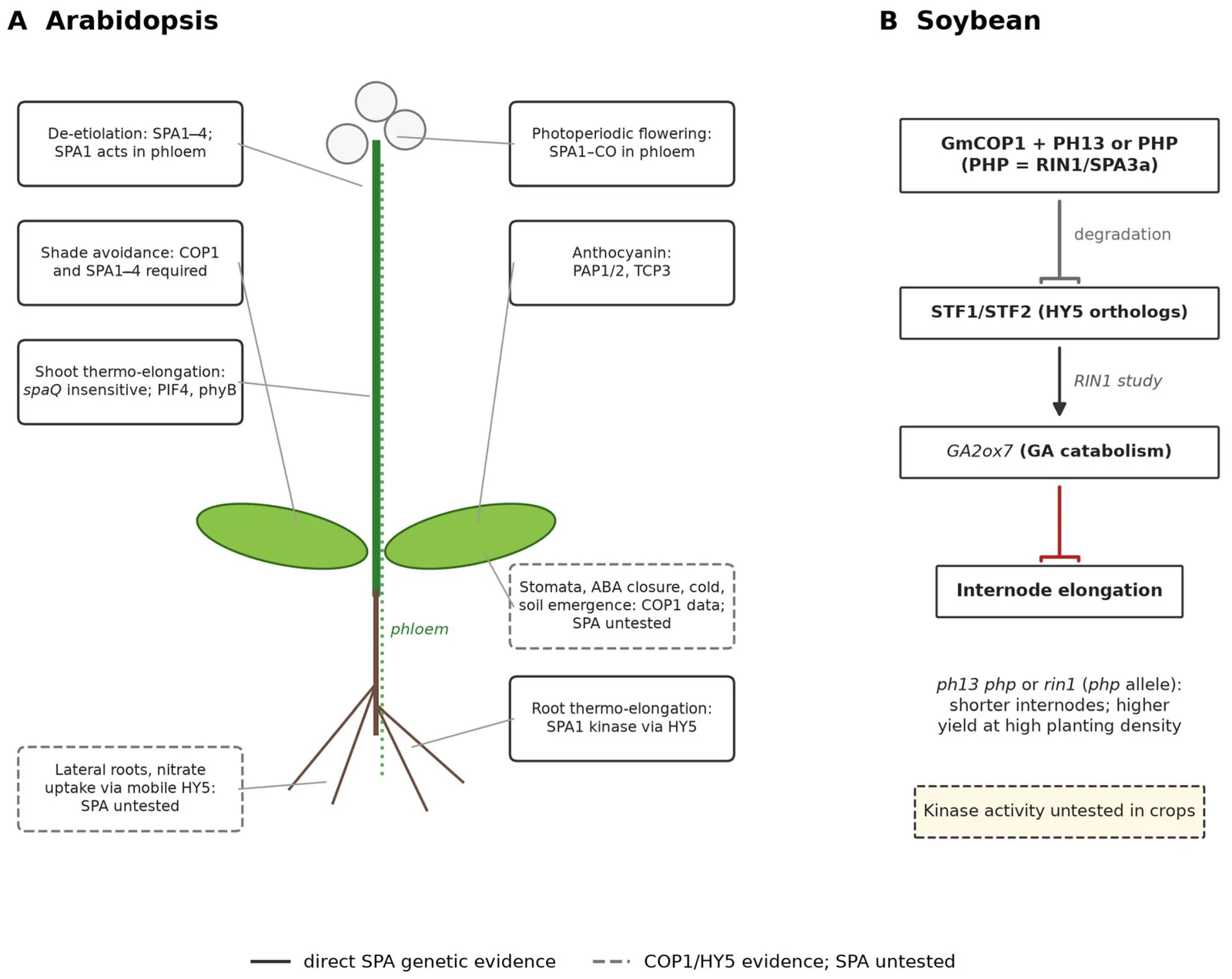
Organ-level map of SPA-dependent processes. (**A**) Processes in Arabidopsis for which SPA involvement has been shown genetically (solid boxes) or has only been inferred from COP1 or HY5 data (dashed boxes). Solid boxes: de-etiolation and phloem action [11,42]; shade avoidance [99]; shoot and root thermo-elongation [16,46]; photoperiodic flowering [44]; anthocyanin accumulation [33,74]. Dashed boxes: lateral root growth and nitrate uptake through mobile HY5 [100,101]; stomatal development and ABA-induced closure, cold acclimation and soil emergence [59,102,103,104,105]. (**B**) In soybean, PH13 and its paralog PHP, also named RIN1 (GmSPA3a), act with GmCOP1 to promote degradation of the HY5 orthologs STF1/STF2. In the RIN1 study, STF1/STF2 act through GA2ox7 to limit gibberellin and internode elongation. ph13 php and rin1 plants have shorter internodes and were found to be associated with higher yield at high planting density in field trials. The kinase activity of these proteins has not been tested [22,23]. GA, gibberellin; ABA, abscisic acid.

This section asks how the mechanisms described in Section 3 and Section 4 shape physiology in different organs and developmental stages. For each process we state whether SPA involvement has been tested genetically or is inferred from COP1 or HY5 data, and Figure 4 maps the processes to organs using the same distinction.

### 5.1. Seedling Photomorphogenesis

De-etiolation is the best-characterized SPA-dependent process. *spaQ* seedlings show constitutive photomorphogenesis in darkness, and *spa* mutants are hypersensitive in light, with SPA1 the main contributor [7,11,27]. SPA proteins act here mainly as COP1 cofactors in darkness (Section 3; Table 3) and contribute the reported SPA-dependent outputs on PIF1 and eIF2α in light (Section 4.3 and Section 4.5). Physiologically, de-etiolation is coordinated across tissues. SPA1 acts mainly from the phloem [42], yet hypocotyl growth arrest and cotyledon opening involve all cell layers. We hypothesize that a mobile signal, such as HY5 or a hormone, communicates SPA activity to other tissues, which could be tested by combining phloem-specific expression of wild-type and kinase-impaired SPA1 with tissue-specific reporters. De-etiolation also requires coordinated changes in transcription and translation, and the SPA-dependent increase in translation efficiency [18], together with the COP1–TOR–RPS6 route [97], raises the possibility that SPA proteins contribute to both arms of this transition.

### 5.2. Shade Avoidance and Plant Architecture

Plants detect neighbors through a lower red-to-far-red ratio (R:FR), which reduces phyB activity and releases PIFs to promote elongation [106]. COP1 and all four SPA genes are essential for hypocotyl and petiole elongation in low R:FR, but not for the accelerated flowering that low R:FR also induces [99]. SPA proteins are therefore required for the growth arm of the shade response but not for its developmental-timing arm. In shade, COP1 re-accumulates in the nucleus [107], and COP1 and PIF signaling converge to drive shade avoidance [108]. COP1 also destabilizes DELLA proteins in response to shade or warmth, independently of changes in gibberellin levels [58], and COP1-mediated stabilization of PIF5, which UVR8 disrupts in sunlight, limits elongation [63]. Whether SPA is required for the DELLA and PIF5 effects has not been tested.

In adult plants, paralog specificity becomes physiologically relevant. SPA3 and SPA4 mainly regulate elongation [27], SPA1 in the phloem and mesophyll controls leaf expansion [42], and COL12, which binds COP1 and SPA proteins, regulates flowering time and plant architecture [73]. In soybean, SPA-like proteins control internode length and thereby link this function to planting density (Section 6).

### 5.3. Thermomorphogenesis

Warm ambient temperatures (for example, 28 °C for *Arabidopsis*) cause hypocotyl and petiole elongation, leaf hyponasty and early flowering, and PIF4 mediates these responses [96]. Temperature enters this network at two points. Phytochrome B acts as a thermosensor, because its reversion from the active Pfr to the inactive Pr form speeds up at higher temperature [109,110]. The COP1 pathway provides a second input. The DET1–COP1–HY5 module regulates *PIF4* transcription [111], DET1 and COP1 are required for PIF4 expression and protein stability, HY5 competes with PIF4 at target promoters [112], and high temperature triggers the nuclear import of COP1, which relieves HY5-mediated growth inhibition [113].

SPA proteins are required for this network to respond to temperature in both shoots and roots. *spaQ* seedlings are thermo-insensitive [16], and *spaQ* roots fail to elongate at high temperature. Wild-type SPA1 largely rescues the root defect, whereas kinase-impaired SPA1 does not, and the authors proposed that SPA1 acts through PIF4 in shoots and through HY5 in roots [46]. This is the first direct evidence for SPA function in roots. In shoots, the route remains unresolved and may involve direct PIF4 phosphorylation, reduced phyB levels, the COP1–HY5 branch, or a combination of these (Section 4.3). At the whole-plant level, day length-dependent COP1 dynamics integrate conflicting seasonal light and temperature cues [114]. Whether light- and clock-regulated SPA abundance (Section 3.4 and Section 5.4) contributes to this seasonal integration is a key open question.

### 5.4. Photoperiodic Flowering and Circadian Regulation

SPA proteins are needed to measure day length. *spa1* mutants flower early in short days but not in long days, and higher-order *spa* mutants flower at about the same time in both photoperiods, so the SPA family is essential for inhibiting flowering in non-inductive short days [44]. In short days, *spa1* mutants have higher *FLOWERING LOCUS T* (*FT*) transcript levels but unchanged *CONSTANS* (*CO*) transcript levels [90], which points to control of CO at the protein level. The mechanism is best understood within the external coincidence model, which combines two controls. The first is transcriptional and is set by the circadian clock. In the morning, CDF1 represses *CO* transcription. In the late afternoon of long days, the clock-controlled proteins FKF1 and GI form a blue light-dependent complex on the *CO* promoter, which targets CDF1 for degradation and so allows *CO* to be transcribed [115]. The second control is post-translational and is set by light: CO protein is stabilized in the light in the evening, but it is degraded by the proteasome in the morning and in darkness [116]. COP1/SPA provides the darkness arm of this second control. SPA1 interacts directly with CO and regulates its stability [44], COP1 ubiquitinates CO and promotes its degradation in darkness [71,72], and CRY2, through its interaction with SPA1, inhibits COP1-dependent CO degradation in blue light [37]. As a result, CO protein accumulates only when *CO* transcription coincides with light, at the end of long days. Further COP1/SPA targets refine this response. COL12 delays flowering and is degraded in darkness in a COP1-dependent manner [73], and TCP3 is degraded preferentially under short days, which delays flowering [33]. SPA1 regulates flowering time mainly from the phloem [42], the tissue in which *CO* and *FT* act.

The circadian clock is connected to SPA at two distinct levels. The first is circadian regulation of SPA. Under constant light, SPA1 protein levels oscillate with a circadian rhythm, whereas they remain unchanged in constant darkness, so SPA1 abundance is controlled by both the clock and light; *SPA1* transcript levels also cycle under constant light [90]. Rhythmic SPA1 abundance could make COP1/SPA activity toward CO dependent on time of day, which could be tested by comparing CO protein profiles in *spa1* plants complemented with rhythmically and with constitutively expressed SPA1. The second level is the contribution of SPA to clock function. In *spa1-3*, the free-running period of *CCA1*, *TOC1*/*PRR1* and *SPA1* transcript rhythms under constant light was slightly shortened [90]. The effect is modest and its mechanism unknown. Because light input sets the period of the clock through phytochrome and cryptochrome pathways [117], and SPA1 negatively regulates phyA signaling [7], the period change may reflect altered light input to the clock rather than a role for SPA1 within the oscillator.

A third connection runs through COP1 and the clock protein GI. COP1 and EARLY FLOWERING 3 (ELF3) together regulate GI stability, and mutations in either gene disturb the rhythmic accumulation of GI, with consequences for both clock function and photoperiodic flowering [60]. Because GI also controls *CO* transcription through the FKF1–GI complex [115], this module places COP1 at both the transcriptional and the post-translational control of CO. Whether SPA proteins participate in COP1–ELF3–GI complexes has not been tested. SPA could therefore act on day-length measurement at three points, namely CO stability (established), clock-regulated SPA1 abundance (reported) and GI turnover (hypothesis). On current evidence, SPA proteins contribute to photoperiodic flowering mainly by enforcing the light requirement for CO protein accumulation, whereas their contribution to circadian timing is measurable but small and mechanistically unresolved.

### 5.5. Anthocyanin and Secondary Metabolism

Anthocyanins accumulate in Arabidopsis only after light exposure, and very high light increases them further. COP1/SPA shapes two physiological features of this response. The first is the light requirement itself. COP1 and SPA proteins interact with PAP1 and PAP2, the MYB activators of anthocyanin biosynthesis, and PAP1 and PAP2 are degraded in darkness in a proteasome- and COP1-dependent manner, so the light requirement results at least partly from light-mediated stabilization of these factors [74]. The second is protection against overaccumulation. *cop1* and *spa* mutants hyperaccumulate anthocyanins under normal light and under excess high light, so COP1/SPA also limits pigment synthesis when light is excessive [118]. TCP3 degradation [33] and blue light-dependent substrate displacement by the CRY1 VP motif [82] add further layers to this control (Table 3; Section 3.3).

Metabolic pathways are strongly enriched among SPA-regulated genes in dark-grown seedlings [21], so SPA control of metabolism probably extends beyond anthocyanins. UV-B-induced flavonoid accumulation depends on UVR8–COP1 signaling [86] and on reorganization of the COP1/SPA complex [83], but an SPA requirement has not been tested. In apple, MdCOP1 degrades the fruit-color regulator MdMYB1 in darkness, which limits red fruit coloration [119]. The contribution of apple SPA homologs has not been examined.

### 5.6. Root Development and Hormone Crosstalk

Roots are usually shaded by soil, but their growth is coordinated with light perceived by the shoot. COP1 controls shoot-to-root auxin transport by regulating *PIN1* transcription in the shoot and the intracellular distribution of PIN1 and PIN2 in the root [120]. HY5 moves from shoot to root, where it activates root *HY5* and nitrate uptake genes and so couples shoot carbon assimilation with root nitrogen acquisition [100], and shoot-perceived low R:FR suppresses lateral root outgrowth through HY5 [101]. The only direct genetic evidence for SPA function in roots concerns root elongation at high temperature (Section 5.3) [46]. Whether shoot-derived mobile HY5 links SPA1 activity in the phloem [42] to lateral root development, nitrate uptake or light-controlled auxin transport remains untested.

SPA proteins and the COP1/SPA module also intersect with several hormone pathways. For gibberellin, COP1 destabilizes DELLA proteins (Section 5.2) [58], and soybean RIN1 acts through *GA2ox7* (Section 6) [23]. For brassinosteroids, COP1/SPA restrains BIN2 (Section 3.2) [64], and BIN2 phosphorylates PIF4 [92]. For jasmonate and abscisic acid (ABA), MYC2 directly regulates *SPA1* expression, and MYC2 and SPA1 act redundantly in darkness but synergistically in light [89]. For auxin, COP1 regulates PIN transport [120], and SPA proteins control PIF4, a major activator of auxin biosynthesis [16,96]; many auxin- and gibberellin-related transcription factors are also deregulated in *spaQ* [21]. Most of these links were established for COP1 or the COP1/SPA complex, and the specific contribution of SPA, and of SPA1 kinase activity, remains to be dissected.

### 5.7. Abiotic and Biotic Stress Contexts

Beyond light and warm temperature, the COP1 module operates in several stress contexts. In each case the evidence concerns COP1 or HY5, and an SPA requirement has not been tested, but each response depends on light-regulated COP1 activity and therefore indicates where SPA could act. During cold acclimation, low temperature stabilizes HY5 by depleting COP1 from the nucleus, and HY5 then promotes cold-induced gene expression and full acclimation [104]. In leaves, COP1 degrades the transcription factors ICE1/SCRM in darkness, so that light promotes stomatal development [59,102], and it promotes ABA-induced stomatal closure by ubiquitinating clade A PP2C phosphatases of the ABI/HAB group and AHG3 [103]. Buried seedlings use COP1 to sense soil depth: COP1 degrades the F-box proteins EBF1 and EBF2 and thereby stabilizes EIN3, whereas mechanical pressure acts through ethylene [105]. In tomato fruit, the E3 ligase SlCOP1-1 stabilizes the transcription factor SlOpaque2 and enhances resistance to *Botrytis cinerea*, an unusual case in which COP1 stabilizes rather than degrades a target [121].

Because SPA proteins are required for full COP1 activity and for its light-dependent inactivation, they may also take part in these responses. Testing *spa* mutants, including paralog-specific combinations, in these assays would show whether the physiological reach of SPA extends beyond light and warm temperature.

## 6. SPA Genes in Crops: Evidence and Limits

Soybean provides the clearest crop evidence (Table 2), and its experimental basis should be stated precisely. *PH13* (Glyma.13G276700) encodes a WD40 protein that the main text of the original study describes as an SPA homolog with kinase, coiled-coil and WD40 domains [22]. A natural variant, *PH13^H3^*, carries a Ty1/Copia-like retrotransposon insertion that removes part of the WD40 domain and weakens the interaction with GmCOP1s. As a result, the HY5 orthologs STF1 and STF2 accumulate and inhibit internode elongation [22]. *PH13^H3^* was strongly selected at high latitudes. The high-density yield benefit was reported for the double mutant of *PH13* and its paralog *PHP* [22]. In a second study from partly overlapping research groups, the short-internode mutant reduced internode 1 (*rin1*) was traced to partial loss of SPA3a (Glyma.12G224600), the same gene that the first study named *PHP*. RIN1 interacts with STF1/STF2 to promote their degradation and controls gibberellin metabolism through *GA2ox7*. In field trials, *rin1* increased grain yield relative to its wild-type elite cultivar under high-density planting while maintaining node and pod number [23].

Both studies therefore concern the same pair of paralogs, PH13 and PHP/RIN1, and together they support three conclusions. First, reducing the activity of SPA-like proteins, through truncation of the WD40 domain or through partial loss of function, shortens soybean internodes and stabilizes STF1/STF2. Second, these alleles were associated with higher yield under high-density planting in the field trials reported, which were conducted in particular genetic backgrounds and environments. Third, the crop evidence supports the COP1 cofactor role of SPA-like proteins. Neither study tested kinase activity or COP1-independent functions. Whether the yield effects hold across environments, latitudes and genetic backgrounds, and whether they can be transferred to other crops, have not yet been reported. Evidence from other crops is limited to heterologous expression. Rice OsSPA1 and maize ZmSPAs modify photomorphogenesis when expressed in *Arabidopsis*, and ZmSPAs also increase plant height [49,53], but loss-of-function alleles have not been characterized in these species.

Crop work on COP1 itself indicates both opportunities and pleiotropic risks. The rice COP1 ortholog PETER PAN SYNDROME (PPS) regulates the juvenile-to-adult and vegetative-to-reproductive phase changes [122]. In apple, MdCOP1 controls fruit coloration through MdMYB1 [119]. In tomato, SlCOP1-1 contributes to fruit resistance to *Botrytis cinerea* [121]. Because COP1 regulates many traits at once, targeting individual SPA paralogs or domains might be more selective than modifying COP1, but this remains to be tested. Table 5 lists research priorities that follow from these studies, together with the evidence on which each is based.

## 7. Conclusions and Priorities for Testing the SPA Hub Model

Since *spa1* was isolated as a suppressor of a weak *phyA* allele [7], the view of SPA proteins has changed from phyA-specific signaling intermediates, to essential COP1 cofactors, to proteins with reported kinase activity. Sorted by evidence level, the current picture is as follows. Several conclusions are established. The four *Arabidopsis* SPA proteins share a tripartite architecture and act partially redundantly with COP1. Their specificity arises mainly from differential expression, tissue context and paralog-specific, NTD-controlled turnover. They are required for full COP1 activity in darkness and for its inactivation by phytochromes and cryptochromes in light, and they regulate de-etiolation, shade-induced elongation, thermomorphogenesis, photoperiodic flowering and anthocyanin accumulation. Other findings are reported but not independently replicated. Recombinant SPA1 phosphorylates PIF1, PIF4, HY5 and eIF2α in vitro, and SPA proteins are required for the phosphorylation of PIF1, HY5 and the eIF2α C-terminus in vivo. Roles of SPA in lateral root development, nitrate uptake, cold acclimation, stomatal regulation, soil emergence and pathogen resistance are inferred from COP1 or HY5 data. SPA-specific output selection and COP1-independent SPA activity remain hypotheses.

Taken together, these findings support the central argument of this review: SPA proteins are best viewed as candidate signal-integrating subunits of the COP1/SPA complex rather than as accessory cofactors. Three features underpin this view (Section 4.7). Phytochrome and cryptochrome inputs dock on SPA domains, CRY2 on the NTD and CRY1 on the WD40 domain. SPA abundance, unlike that of COP1, is regulated by light, the circadian clock and MYC2, and SPA2 undergoes paralog-specific, NTD-dependent turnover. Additionally, the only other biochemical output reported for the complex, kinase activity, resides in SPA1. The model also has clear limits. The complex, not SPA alone, executes most outputs; kinase activity is reported for SPA1 only and has been neither independently replicated nor catalytically validated; and neither output selection nor COP1-independent SPA activity has been demonstrated.

Four experiments would test the model most decisively, in order of priority. (1) Independent replication of SPA1 kinase activity, using protein expressed in a heterologous system, purified to homogeneity and assayed alongside catalytically inactive variants purified in parallel, ideally supported by a structure of the NTD. This is a precondition for the other tests. (2) Catalytic validation in planta, using several independent catalytic-site substitutions that fold correctly, bind COP1 and photoreceptors normally and are as stable as wild-type SPA1, each expressed in *spaQ*. (3) Site-resolved in vivo phosphoproteomics of PIF1, PIF4, HY5 and eIF2α in *spaQ*, *ck2* and *ppk* backgrounds, combined with expression of kinase-impaired SPA1 in *spaQ phyB* to separate direct PIF4 phosphorylation from the phyB route. (4) Tests of COP1 independence and output selection, using SPA variants that retain kinase activity but cannot bind COP1, and measuring the kinase and E3 outputs of the same SPA pool under the same conditions. Testing whether SPA2–SPA4 and crop SPA proteins are active kinases would show how general the second output is. If these experiments confirm a catalytically active, light-regulated SPA kinase whose output is coupled to or selected against the E3 output, SPA proteins would qualify as signaling hubs in their own right; if not, they would remain essential, signal-receiving cofactors of COP1.

## Figures and Tables

**Table 1:** Comparison of *Arabidopsis* SPA paralogs. “Not tested” means that we found no published data.

Property	SPA1 (AT2G46340)	SPA2 (AT4G11110)	SPA3 (AT3G15354)	SPA4 (AT1G53090)	Refs.
Protein length (residues)	1029	1036	845	794	[43]
Main role in dark-grown seedlings	Major	Major	Minor	Minor	[11,27]
Main role in light-grown seedlings	Major (all light qualities)	Largely inactivated by light; residual activity	Contributes	Contributes	[10,40,41]
Adult elongation growth	Minor	Minor	Major	Major	[27]
Photoperiodic flowering	Major (phloem)	Minor	Contributes	Contributes	[42,44]
Light-induced degradation	Weak	Rapid (5–15 min), COP1-dependent, requires NTD and WD40	Stable in light	Stable in light	[39,40,45]
Kinase activity tested	Yes (in vitro; kinase-impaired variant in vivo)	Not tested	Not tested	Not tested	[15,17,18]
Tissue of action	Phloem sufficient for seedling and flowering phenotypes; required for root thermomorphogenesis (*spaQ* complementation)	Not tested	Not tested	Not tested	[42,46]

**Table 2:** Functionally characterized SPA and SPA-like genes outside *Arabidopsis thaliana*. N, evidence from the native species; H, heterologous expression in *Arabidopsis*.

Species	Gene(s)	Gene Identifier	Evidence	Main Finding	Refs.
*Marchantia polymorpha* (liverwort)	*MpSPA*	Mp3g25460	N (biochemistry, genetics)	MpSPA promotes MpCOP1-mediated ubiquitination of MpHY5; MpCRY does not inhibit it	[52]
*Physcomitrium patens* (moss)	*PpSPAa*, *PpSPAb*	Pp1s59_66V6.1 (*PpSPAa*); Pp1s30_295V6.1 (*PpSPAb*)	N (knockouts); H	Smaller gametophores in light, continued growth in darkness, normal etiolation; co-action with COP1 and cryptochrome	[49,50,51]
*Oryza sativa* (rice)	*OsSPA1*; an *OsSPA3/4*-like gene	LOC_Os05g49590 (*OsSPA1*); LOC_Os01g52640 (*OsSPA3/4*-like)	H	OsSPA1 rescues dark-grown but not light-grown or adult *spa* phenotypes	[49]
*Zea mays* (maize)	*ZmSPAs*	Zm00001eb344140, Zm00001eb296410, Zm00001eb154420, Zm00001eb361760 (*ZmSPA1*–*ZmSPA4*)	H	Modulate photomorphogenesis; promote plant height	[53]
*Glycine max* (soybean)	*PH13* and paralog *PHP*	Glyma.13G276700 (*PH13*); Glyma.12G224600 (*PHP*)	N (natural variation; mutants; field trials)	Retrotransposon-truncated PH13 WD40 weakens GmCOP1 binding; STF1/2 accumulate; shorter internodes; the high-density yield benefit was reported for the *ph13 php* double mutant	[22]
*Glycine max* (soybean)	*RIN1*/*GmSPA3a* (same gene as *PHP*)	Glyma.12G224600	N (mutant; field trials)	Partial-loss allele shortens internodes via STF1/2–*GA2ox7*; higher grain yield than the wild type under high-density planting in field trials	[23]

**Table 4:** Evidence for SPA1 kinase activity toward individual substrates. Level 1, phosphorylation of the substrate by recombinant SPA1 in vitro; Level 2, SPA-dependent phosphorylation of the substrate in vivo (reduced in *spaQ*); Level 3, failure of a kinase-impaired but otherwise intact SPA1 variant to restore phosphorylation or function in *spaQ*. All kinase assays used SPA1, and none of the findings has yet been replicated by an independent laboratory. “Not reported” means that we found no such data in the cited studies.

Substrate	Level 1: In Vitro	Level 2: In Vivo SPA Dependence	Level 3: Kinase-Impaired SPA1 In Vivo	Sites and Substrate Variants	Other Kinases Reported	Refs.
PIF1	Yes	Yes; light-induced phosphorylation and degradation reduced in *spaQ*	Partial; SPA1^R517E^ defective in PIF1 degradation; COP1 binding of the variant not reported	SPA sites not reported	CK2 (seven sites); PPKs and phytochrome shown for PIF3	[15,36,75,93,94]
PIF4	Yes	Not reported. SPA-dependent PIF4 phosphorylation in vivo has not been reported; PIF4 accumulation at warm temperature requires SPA, and phyB is stabilized in *spaQ*	Partial, for PIF4 protein level only: kinase-impaired SPA1 fails to restore PIF4 accumulation and thermo-elongation in *spaQ*; PIF4 phosphorylation was not assessed	Not reported	BIN2	[16,92]
HY5	Yes	Yes; phospho-HY5 absent in *spaQ*	Partial; SPA1^R517E^ (mSPA1) fails to rescue root thermomorphogenesis of *spaQ*	Ser36; HY5^S36A^ tested	PPK1 (Thr55, Ser56, Ser60); CK2-like activity proposed	[15,17,46,65,91]
eIF2α (C-terminus)	Yes	Yes; light-induced C-terminal phosphorylation and translation efficiency reduced in *spaQ*	Partial; kinase-mutant SPA1 (mSPA1) fails to complement translational changes	C-terminal Ser/Thr; non-phosphorylatable variant tested	Not reported for these sites	[18]

**Table 5:** Research priorities for evaluating SPA genes in crops.

Priority	Current Evidence	Suggested Approach	Traits to Monitor
1. Survey SPA allelic diversity in crop germplasm	Field and crop mutant (soybean only); natural PH13 variation was selected at high latitudes, and partial loss of RIN1 was associated with higher yield at high density [22,23]	Identify WD40-truncating and regulatory variants of SPA3/4-type paralogs in soybean, maize and other crop pangenomes	Internode length, lodging, yield per area
2. Test partial and domain-specific alleles	Arabidopsis and crop mutant; null *spaQ* and *cop1* phenotypes are strongly pleiotropic, whereas *PH13^H3^* and *rin1* are partial alleles [11,22,23]	Base editing or small in-frame deletions in the WD40 domain; promoter edits	Seedling vigor, flowering time
3. Test whether the kinase arm operates in crops	Untested; SPA1 kinase activity has been reported only in Arabidopsis	Kinase-impaired alleles, after the Arabidopsis controls in Section 4.2	PIF levels, thermo-elongation
4. Evaluate performance across environments	Arabidopsis (photoperiodic flowering and thermomorphogenesis); field data limited to the reported soybean trials	Multi-environment, multi-year field trials across latitudes	Flowering date, heat-induced elongation, yield stability
5. Check stress trade-offs	Untested for SPA; COP1 acts in cold, ABA and pathogen responses	Stress assays on candidate lines	Cold tolerance, water use, disease

## Data Availability

No new experimental data were generated in this study. The domain boundaries in Figure 1 were derived from publicly available protein sequences (UniProtKB [43]) and structures (Protein Data Bank entry 6QTO) as described in the figure legend.

## Abbreviations

The following abbreviations are used in this manuscript:

ABA	Abscisic acid
AGI	Arabidopsis Genome Initiative (locus identifier)
BBX	B-box protein
bHLH	Basic helix-loop-helix
BIN2	BRASSINOSTEROID-INSENSITIVE 2
bZIP	Basic leucine zipper
CC	Coiled-coil
CCA1	CIRCADIAN CLOCK ASSOCIATED 1
CCE	Cryptochrome C-terminal extension
CDF1	CYCLING DOF FACTOR 1
CK2	Casein kinase 2
CO	CONSTANS
COL12	CONSTANS-LIKE 12
COP1	CONSTITUTIVELY PHOTOMORPHOGENIC 1
CRY	Cryptochrome
cryo-EM	Cryo-electron microscopy
CUL4	CULLIN4
DDB1	DAMAGED DNA BINDING PROTEIN 1
DET1	DE-ETIOLATED 1
EBF1/2	EIN3-BINDING F-BOX PROTEIN 1/2
eIF2α	Eukaryotic translation initiation factor 2α
EIN3	ETHYLENE INSENSITIVE 3
ELF3	EARLY FLOWERING 3
FKF1	FLAVIN-BINDING, KELCH REPEAT, F-BOX 1
FR	Far-red light
FT	FLOWERING LOCUS T
GA	Gibberellin
GA2ox7	GIBBERELLIN 2-OXIDASE 7
GI	GIGANTEA
HFR1	LONG HYPOCOTYL IN FAR-RED 1
HY5	ELONGATED HYPOCOTYL 5
HYH	HY5 HOMOLOG
ICE1/SCRM	INDUCER OF CBF EXPRESSION 1/SCREAM
LAF1	LONG AFTER FAR-RED LIGHT 1
MYB	MYB-domain transcription factor
MYC2	MYC2 bHLH transcription factor
NTD	N-terminal domain
PAP	PRODUCTION OF ANTHOCYANIN PIGMENT
PDB	Protein Data Bank
Pfr/Pr	Far-red-absorbing (active)/red-absorbing (inactive) phytochrome form
PH13	PLANT HEIGHT 13
PHP	PH13 paralog
phy	Phytochrome
PIF	PHYTOCHROME INTERACTING FACTOR
PIN	PIN-FORMED auxin efflux carrier
PP2C	Protein phosphatase 2C (clade A: ABI/HAB, AHG3)
PPK	PHOTOREGULATORY PROTEIN KINASE
PPS	PETER PAN SYNDROME (rice COP1 ortholog)
R:FR	Red-to far-red ratio
RIN1	REDUCED INTERNODE 1
RING	Really Interesting New Gene (zinc-finger domain)
RPS6	RIBOSOMAL PROTEIN S6
SPA	SUPPRESSOR OF PHYA-105
*spaQ*	*spa1 spa2 spa3 spa4* quadruple mutant
STF	SOYBEAN TGACG-MOTIF BINDING FACTOR (soybean HY5 orthologs)
TCP	TEOSINTE BRANCHED1/CYCLOIDEA/PCF
TF	Transcription factor
TOC1/PRR1	TIMING OF CAB EXPRESSION 1/PSEUDO-RESPONSE REGULATOR 1
TOR	TARGET OF RAPAMYCIN
Ub	Ubiquitin
UVR8	UV RESISTANCE LOCUS 8
VIL1	VERNALIZATION INSENSITIVE 3-LIKE 1
VP	Valine–proline motif
WD40	Tryptophan–aspartate 40-residue repeat

## Appendix A

**Table A1:** Gene identifiers for the SPA and COP1 genes and for the targets, photoreceptors, kinases and regulators shown in Table 1, Table 2, Table 3 and Table 4 and Figure 1, Figure 2, Figure 3 and Figure 4. Arabidopsis genes are given as AGI codes (TAIR); crop and bryophyte genes as reported in the cited studies or in the respective genome databases.

Gene or Protein	Identifier	Gene or Protein	Identifier
SPA1	AT2G46340	PIF1	AT2G20180
SPA2	AT4G11110	PIF3	AT1G09530
SPA3	AT3G15354	PIF4	AT2G43010
SPA4	AT1G53090	PIF5	AT3G59060
COP1	AT2G32950	eIF2α	AT5G05470
CUL4	AT5G46210	BIN2	AT4G18710
DDB1A	AT4G05420	PPK1	AT3G13670
PHYA	AT1G09570	MYC2	AT1G32640
PHYB	AT2G18790	GI	AT1G22770
CRY1	AT4G08920	ELF3	AT2G25930
CRY2	AT1G04400	FT	AT1G65480
UVR8	AT5G63860	VIL1	AT3G24440
HY5	AT5G11260	ICE1/SCRM	AT3G26744
HYH	AT3G17609	GAI	AT1G14920
HFR1	AT1G02340	RGA	AT2G01570
LAF1	AT4G25560	RGL1	AT1G66350
BBX21	AT1G75540	RGL2	AT3G03450
BBX22	AT1G78600	RGL3	AT5G17490
BBX24	AT1G06040	*Glycine max* PH13	Glyma.13G276700
BBX28	AT4G27310	*Glycine max* PHP (= RIN1/SPA3a)	Glyma.12G224600
CO	AT5G15840	*Marchantia polymorpha* MpSPA	Mp3g25460
COL12	AT3G21880	*M. polymorpha* MpCOP1	Mp5g12010
TCP3	AT1G53230	*Physcomitrium patens* PpSPAa, PpSPAb	Pp1s59_66V6.1, Pp1s30_295V6.1
PAP1/MYB75	AT1G56650	*Oryza sativa* OsSPA1, OsSPA3/4-like	LOC_Os05g49590, LOC_Os01g52640
PAP2/MYB90	AT1G66390	*Zea mays* ZmSPA1, ZmSPA2	Zm00001eb344140, Zm00001eb296410
CK2, PPK2–PPK4	Multigene families; see [75,93]	*Z. mays* ZmSPA3, ZmSPA4	Zm00001eb154420, Zm00001eb361760
